# Phytoceuticals in Acute Pancreatitis: Targeting the Balance between Apoptosis and Necrosis

**DOI:** 10.1155/2018/5264592

**Published:** 2018-03-04

**Authors:** Laura Gaman, Dorin Dragos, Adelina Vlad, Georgiana Catalina Robu, Mugurel Petrinel Radoi, Laura Stroica, Mihaela Badea, Marilena Gilca

**Affiliations:** ^1^Faculty of General Medicine, Carol Davila University of Medicine and Pharmacy, B-dul Eroilor Sanitari No. 8, Sector 6, 76241 Bucharest, Romania; ^2^Nephrology Clinic, University Emergency Hospital Bucharest, 050098 Bucharest, Romania; ^3^National Institute of Neurology and Neurovascular Diseases, Bucharest, Romania; ^4^Transilvania University of Brașov, Brașov, Romania

## Abstract

Despite recent advances in understanding the complex pathogenesis of pancreatitis, the management of the disease remains suboptimal. The use of phytoceuticals (plant-derived pleiotropic multitarget molecules) represents a new research trend in pancreatology. The purpose of this review is to discuss the phytoceuticals with pancreatoprotective potential in acute pancreatitis and whose efficacy is based, at least in part, on their capacity to modulate the acinar cell death. The phytochemicals selected, belonging to such diverse classes as polyphenols, flavonoids, lignans, anthraquinones, sesquiterpene lactones, nitriles, and alkaloids, target the balance between apoptosis and necrosis. Activation of apoptosis via various mechanisms (e.g., inhibition of X-linked inhibitor of apoptosis proteins by embelin, upregulation of FasL gene expression by resveratrol) and/or inhibition of necrosis seem to represent the essential key for decreasing the severity of the disease. Apart from targeting the apoptosis/necrosis balance, the phytochemicals displayed other specific protective activities: inhibition of inflammasome (e.g., rutin), suppression of neutrophil infiltration (e.g., ligustrazine, resveratrol), and antioxidant activity. Even though many of the selected phytoceuticals represent a promising therapeutic alternative, there is a shortage of human evidence, and further studies are required to provide solid basis to justify their use in the treatment of pancreatitis.

## 1. Introduction

Despite recent advances in understanding the pathogenesis of pancreatitis and identification of new therapeutic solutions, the management of the disease, especially that of severe acute pancreatitis (SAP), remains suboptimal, and this clinical form is still associated with a high mortality rate [1–4]. Several studies reported values between 25% and 35% in case of pancreatitis complicated with persistent organ failure [5, 6].

Pancreatitis is an inflammatory disease associated also with important parenchymal cell death [7]. It involves escape of abnormally activated intra-acinar protease zymogens and lipase, into the interstitium of the pancreas, causing autodigestion of the pancreatic tissue [8, 9]. It is often complicated with systemic inflammation and multiorgan dysfunction syndrome.

The current therapeutic guidelines for acute pancreatitis (AP) include the following: intravenous fluid replacement, dietary changes, analgesics, inhibitors of pancreatic secretion (somatostatin and its analogue, octreotide), L-arginine, calcium ion antagonists, and different inflammatory mediator inhibitors [10–13].

Unfortunately, the use of standard drugs in acute pancreatitis is still disappointing. Also, the available drugs (somatostatin and octreotide) have a short half-life and their clinical efficiency is limited [14, 15]. Hence, there is renewed interest in phytomedicines, which lack severe adverse effects and may have benefits not only regarding the symptoms but also regarding the disease evolution.

Various animal models of pancreatitis have been used to study the properties of both medicinal plants extracts and individual phytochemicals, used alone or in combination (Table 1).

The purpose of this review is to summarize the available scientific information obtained from medical databases and literature on phytochemicals that have been reported to have therapeutic potential in acute pancreatitis, whose efficacy is based, at least in part, on their capacity to modulate the acinar cell death.

## 2. Methodology

A literature search was performed using the following phrases “phytochemicals AND acute pancreatitis OR pancreas”, “medicinal plant OR herb AND acute pancreatitis OR pancreas”, and “specific phytochemical name AND pancreatitis or pancreas” (e.g., resveratrol AND pancreatitis OR pancreas), in PubMed and Elsevier database.

*Selection Criteria*. Relevant studies (*in vitro* and/or on animal models and/or on human subjects) found in PubMed and Elsevier databases were collected regardless of study design, language, year of publication, or publication status. Standardized criteria were utilized for selection. At least one* in vitro* or animal study investigating the effect of the phytocompound on the apoptosis of pancreatic acinar cells in an experimental model of acute pancreatitis was inclusion criterion.

*Data Analysis*. Data extraction and analysis were performed by professionals conducting medical research or clinical work at academic level. The most significant results for our subject were retrieved. Data were verified by a second author.

Relevant phytochemicals are presented in an alphabetical order: artemisinin, baicalin, crambene, curcumin, embelin, emodin, hesperidin, honokiol, ligustrazine, magnolol, naringin, nordihydroguaiaretic acid, resveratrol, rhein, and rutin.

## 3. Key Aspects of Pancreatitis Pathogenesis

### 3.1. Inflammatory Response in Pancreatitis

A plenty of studies showed that proinflammatory cytokines (e.g., TNF-*α*, IL-1*β*, and IL-6) are key factors in AP pathogenesis and progression towards systemic complications [27–29]. The stress kinases ERK (extracellular signal-regulated kinase), JNK (c-jun N-terminal kinase), and p38, which are members of the mitogen-activated protein kinase (MAPK) cascade, seem to play an important role in pancreatic overproduction of these proinflammatory cytokines [30, 31]. Therefore, inhibitors or activators of ERK, p38, and JNK can modulate the tissue damage in animal models of pancreatitis [32].

Other factors (e.g., platelet activating factor (PAF,) pancreatic polypeptide, peptide YY, substance P, intestinal gut dysfunction, and endotoxinemia) were cited as contributors to the pathogenesis of pancreatitis and of its complications [33–36]. For instance, PAF, an inflammatory mediator involved in the processes leading to pancreatitis, has ubiquitous receptors (on pancreatic islet cells [37], pancreatic microvascular endothelium [38], bronchia epithelial cells [37], and leukocytes [39]) and therefore could amplify both the local and the systemic inflammatory response [36, 40].

Several studies suggested that upregulation of toll-like receptor 4 (TLR4), constitutively expressed in pancreas, as well as in other tissues (e.g., leukocytes, endothelial cells, lung, kidney, and heart) may play a role in the local pancreatic injury and in the systemic inflammatory response, through stimulation of cytokine synthesis [41–44].

In addition to acinar cells and lymphocytes, there are also a few other cell categories that represent key actors in the scenario of pancreatitis pathogenesis. Several families of macrophages, which are activated during different stages of AP, are involved in the disease progression: peritoneal macrophages (pMΦs), Kupffer cells, and alveolar macrophages [45, 46]. Pancreatic stellate cells (PSCs), activated as a response to pancreatic injury or inflammation via transforming growth factor-*β* (TGF-*β*), are multifunctional cells which play a crucial profibrogenic role in chronic pancreatitis. It is already established that PSCs are involved in inflammation, apoptosis, insulin expression, and exocrine function in the pancreas [47].

### 3.2. Parenchymal Cell Death in Pancreatitis

Despite significant progress in revealing the molecular mechanisms of inflammatory response in pancreatitis, little is known about the pathways of parenchymal cell death [7].

Apoptosis and necrosis represent the two main types of cell death: the “good” (environment-friendly) and the “bad” (environment-spoiling), respectively, because apoptosis, by contrast to necrosis, does not elicit an inflammatory response and hence does not injure the surrounding cells [48]. Moreover, apoptosis seems to generally limit the inflammatory cascade, having an active protective effect [49, 50]. These may explain why enhancing apoptosis reduces the severity of AP [51, 52] (the apoptosis-to-necrosis ratio being high in mild AP and low in SAP [53]). Interestingly, the mechanisms by which somatostatin analogue octreotide relieves acute pancreatitis in mice seem to be correlated with the induction of apoptosis in pancreatic acinar cells [54].

Nevertheless, apoptosis role is dual, being dependent on its intensity. Pancreatic acinar cells undergoing apoptosis, as well as necrosis, can release damage-associated molecular patterns (DAMPs), such as histones, DNA, and heat shock proteins (HSP). Apoptotic cell remnants are rapidly phagocytosed [55], while necrotic cell remnants are less efficiently removed [7, 56]. When the rate of DAMPs phagocytosis is surpassed by the rate of apoptosis, the accumulated DAMPs, which have proinflammatory effects, may aggravate the pancreatic injury [49].

There are two main distinct signalling pathways leading to apoptosis:

*Extrinsic* pathway: initiated by the activation of death receptors, like TNF receptor or Fas receptor, and mediated by caspase-8, which eventually activates caspase-3

*Intrinsic* pathway (a.k.a. mitochondrial pathway): initiated by reactive oxidative species (ROS), hypoxia, and so forth; it requires the disruption of the mitochondrial membrane, which leads to the release of cytochrome c, known to be involved in the activation of caspase-9, which further activates caspase-3 [7].

Caspases, cysteine proteases that act in cascade, are classified into two categories: initiator caspases (e.g., caspase-2, caspase-8, caspase-9, and caspase-10) and effector caspases (e.g., caspase-3, caspase-6, and caspase-7), depending on their position and role in apoptosis pathway [57].

The permeabilization of the outer mitochondrial membrane responsible for cytochrome c release is regulated by the Bcl-2 family members, which are either antiapoptotic (e.g., Bcl-2) or proapoptotic (e.g., Bax, Bak) [58]. Inhibitor of apoptosis proteins (IAPs) represent another category of apoptosis regulatory agents that bind to and inhibit caspases. For instance, X-linked IAP (XIAP) was found to be involved in the caspase blockade in pancreatitis [7], and its deletion decreased the severity of AP via regulation of cell death (enhanced apoptosis and reduced necrosis in pancreatic acinar cells) and nuclear factor-*κ*B activity [59]. High doses of colecistokinin-8 (CCK-8) induced apoptosis in isolated pancreatic acinar cells, by releasing cytochrome c and consequent activation of caspase-9, caspasese-3, and caspase-8 [60].

Other apoptosis pathways seem to be involved in pancreatitis pathogenesis. One of them is the p53-mediated apoptosis pathway, which involves formation of PIDDsome, a complex consisting of PIDD (p53-induced protein with death domain), RAIDD (RIP-associated ICH-1/CED-3 homogenous protein with death domain), and procaspase-2 [61]. PIDD expression is controlled by p53 [62]. PIDDsome assembling results in caspase-2 activation [63], which directly activates effector caspases [64], leading to apoptosis [65]. Nevertheless, there are studies indicating that apoptosis may develop without caspase activation. These alternative pathways seem to be mediated by mitochondrial factors, like apoptosis-inducing factor (AIF), endonuclease G (endoG), and so forth [66], but it is not yet clarified if these pathways are involved in pancreatitis pathogenesis.

There are also two distinct signalling pathways leading to necrosis:

*Extrinsic* pathway: initiated by the activation of death receptors, similarly to apoptosis extrinsic pathway, but differently mediated, by TNFR1 complex II and receptor-interacting protein kinases (RIPKs)

*Intrinsic* pathway (initiated by severe cellular stress producing a mitochondrial dysfunction that eventually leads to ATP depletion and increased oxidative stress) [7].

**Figure 1:**
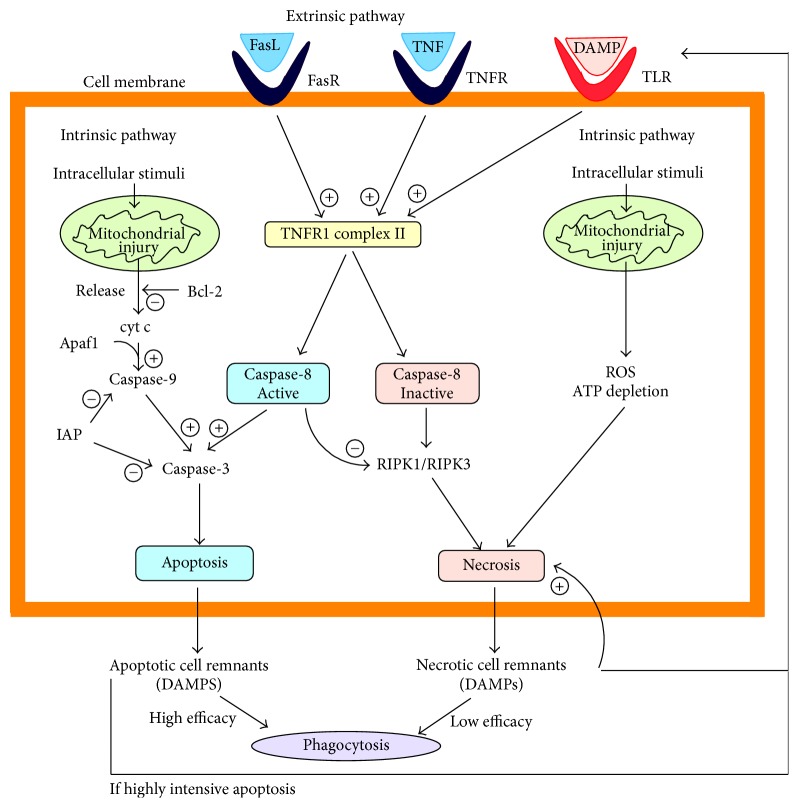
Mechanisms underlying the balance between apoptosis and necrosis in acute pancreatitis (FasL: Fas ligand, FasR: Fas receptor, IAP: inhibitor of apoptosis proteins, DAMP: damage-associated molecular patterns, RIPK: receptor-interacting protein kinases, TLR: toll-like receptors, and TNFR: TNF receptor).

Several members of RIPK family, such as RIPK1 and RIPK3, contribute to parenchymal cell death through necrosis in pancreatitis [67, 68]. It is already established that the activity of RIPK3 is correlated with responsiveness to TNF-alpha induced necrosis [67]. As a consequence of cell lysis, the intracellular content is released in the intercellular matrix, eventually reaching systemic circulation, and DAMPs may trigger local and systemic inflammation [68]. Toll-like receptors (TLRs) sense endogenous danger signals, represented by DAMPs, and mediate the aggravation of pancreatic injury and inflammatory response (Figure 1).

The major pathway of apoptosis is generally considered to be the intrinsic one, in contrast with necrosis main modality, which is mostly extrinsic [52]. Necrosis is either spontaneous or tightly regulated. The best investigated form of regulated necrosis is called necroptosis [69] and is mediated by the receptor-interacting protein kinases RIPK1/RIPK3/mixed lineage kinase domain-like pathway [67]. Interestingly, necroptosis is the major form of acinar cell death in several experimental models of pancreatitis, and its blockage not only might prevent pancreatitis but also might decrease the severity, when this is already established [69, 70].

There are other ways of cell death, like pyroptosis, which is a form of highly inflammatory lytic programmed cell death. Inflammasomes, innate immune system receptors and sensors [71], activate caspase-1 or caspase-11/4/5, leading eventually to the formation of large pores in the membrane, responsible for cell swelling and membrane rupture [69, 72]. When the inflammasome pathway is impaired (e.g., genetic deletion of various components such as TLR9), the cell death rate and inflammation in pancreatitis are decreased [73, 74].

### 3.3. Calcium and Cell Death in Acute Pancreatitis

It has been demonstrated that Ca^2+^ elevation in the acinar cells cytoplasm leads to the intracellular activation of digestive enzymes and the ensuing physiopathological cascade characteristic for AP. Moreover, the pattern of Ca^2+^ elevation (sustained high level versus fluctuating lower level) may influence the severity of AP [the normal one being (very) low (i.e., normal) level, fluctuating under the influence of various physiologic stimuli, which elicit short-lived increases, sufficient only to drive the extracellular release of the digestive enzymes, but insufficient in magnitude and duration for their activation]. A major stressor infringing upon the acinar cells provokes a sustained, marked increase in cytosolic Ca^2+^, which leads to persistent mitochondrial depolarization [possibly by opening the mitochondrial permeability transition pore (MPTP)]. This results in a drastic ATP shortage that knocks out the energy-hungry Ca^2+^ pumps, leading to Ca^2+^ accumulation in the cytoplasm [with simultaneous depletion of the endoplasmic reticulum (ER)], inappropriate uncontrolled activation of digestive enzymes that wreak havoc in the cell, culminating with Ca^2+^-dependent necrosis. On the other hand, a more subdued aggression elicits a lower level and undulating increase in cytosolic Ca^2+^, leading to ephemeral MPTP opening and hence to only partial mitochondrial depolarization, ROS generation, and activation of the intrinsic (mitochondrial) pathway of apoptosis. Thus the cell's death is peaceful and modestly consequential [75]. Preventing Ca^2+^ from piling up in the cytosol may seem a promising strategy for mitigating AP.

There are differences between various animal models of pancreatitis, in terms of necrosis/apoptosis ratio and caspase activation degree: the rat model of cerulein pancreatitis is characterized by relatively high apoptosis and low necrosis as well as strong activation of caspases, while the mouse model by low apoptosis and high necrosis associated with a lack of caspase activation [7]. The necrosis/apoptosis ratio was 120-fold greater in the mouse model than in the rat model. Caspase inhibition worsened necrosis (resulting in higher amylase and lipase) and inflammatory cell infiltration in rat cerulein pancreatitis, while caspase activation decreased necrosis and normalized pancreatic histology in mouse cerulein pancreatitis, suggesting that caspases are involved not only in induction of apoptosis but also in protection against necrosis [7].

### 3.4. Cross-Talk between Apoptosis, Necrosis, and Inflammatory Pathways in Acute Pancreatitis

It is worth mentioning that there is a significant cross-talk between the apoptosis and necrosis signalling pathways involved in pancreatitis pathogenesis:

1. Cross-talk between extrinsic and intrinsic pathways of apoptosis, such as indirect activation of caspase-8 via the intrinsic pathway, following activation of caspase-3 [76], or activation of BH3 interacting-domain death agonist, a proapoptotic member of the Bcl-2 protein family, by caspase-8 dependent cleavage (extrinsic apoptotic pathway), resulting in cytochrome c release (intrinsic apoptotic pathway) [52]
2. Cross-talk between apoptosis and necrosis signalling pathways, such as activation of both pathways through death receptors by the same death ligands (e.g., binding of TNF-*α* to TNFR) [52] or inactivation of RIPK (necrosis pathway) through proteolytic cleavage by caspase-8 (apoptosis pathway) [77].

### 3.5. Oxidative Stress and Pancreatitis

Oxidative stress is an important pathogenic factor placed at the crossroads between apoptosis, necrosis, and inflammation pathways.

Pancreatic islet cells showed lower levels of antioxidant enzymes and represent a target for heavy metals accumulation (e.g., cadmium); therefore pancreas is more susceptible to oxidative stress than other tissues and organs [78–80]. Pancreas is also highly prone to injury consequent to ischemia produced by various mechanisms (e.g., decreased blood flow induced by ethanol, specific microcirculatory anatomy, and microvascular changes due to acute injuries), followed by reperfusion (which is associated with an increased synthesis of ROS and inflammatory reaction) [81, 82].

Oxidative stress leads to the activation of nuclear factor kappa-light-chain-enhancer of activated B cells (NF-*κ*B) and PI3K/AKT signalling pathways which is also critically involved in generating the inflammatory side of pancreatitis as well as its complications [83–86]. NADPH oxidase activation and increased synthesis of free radicals represent other contributors to the pathogenesis of pancreatitis. The degree of NADPH oxidase activity was positively correlated to the severity of pancreatic oxidative alteration in AP [87].

## 4. Proapoptotic Phytochemicals with Protective Effects in Pancreatitis

Phytoceuticals represent an attractive therapeutic alternative in pancreatitis, more and more patients, as well as scientists, focusing either their personal or scientific interest on the potential benefits of these bioactive plant-derived products (see Table 2). Interestingly, a recent cross-sectional survey of consecutive outpatients seen at a Pancreas Center found that 44% of the patients with pancreatic disorders used complementary and alternative medicines (CAM) (medicinal plants representing 28% of the total CAM), the percentage being even higher (47%) in patients with previous acute pancreatitis [88].

The purpose of this review was to elaborate a hypothesis concerning the potential efficacy of phytoceuticals in acute pancreatitis, which might be based on multitargeting the balance between apoptosis and necrosis, as well as inflammation and oxidative stress.

### 4.1. Artemisinin and Its Derivatives

Artemisinin is a sesquiterpene lactone identified in sweet wormwood (*Artemisia annua*, fam. Asteraceae), in the 1970s, by a Chinese group led by Tu et al. [135]. Its discovery was awarded Nobel Prize in 2015, due to its excellent contribution to human health, having mainly antimalarial but also other important bioactivities (e.g., anti-inflammatory, immunoregulatory, and anticancer bioactivities) [136–139]. Its derivatives such as artesunate, hydroartemisinin, artemether, and arteether have been used to treat malaria in clinical settings.

#### 4.1.1. *In Vitro* Studies

*Inflammation.* Artesunate, a water-soluble hemisuccinate derivative of dihydroartemisinin, substantially inhibited the expression of IL-1*β*, IL-6, TLR4, and NF-*κ*B p65 in the pancreatic acinar cells treated with lipopolysaccharide (LPS), but it did not significantly influence the TNF-*α* release [90].

#### 4.1.2. Animal Studies

*Apoptosis versus Necrosis*. Artemisinin relieved the severity of inflammation in cerulein-induced AP in Wistar rats, by increasing the number of apoptotic cells and caspase-3 activity, while reducing the number of necrotic cells and the level of serum amylase [89]. In another study, the SD rats treated with various doses of artesunate showed significantly milder parenchymal necrosis and hemorrhage than the SAP group [90].

*Inflammation*. Artemisinin decreased pancreatic edema and inflammatory cell infiltration, NF-*κ*B-activation, MIP-1*α* protein, myeloperoxidase (MPO), and IL-1*β* mRNA in cerulein-induced AP of Wistar rats [89]. Artesunate was also found to increase the survival of rats with 3.5% sodium taurocholate-induced SAP, ameliorating their pancreatic histological alterations (necrosis and hemorrhages), decreasing serum amylase and lipase activities, and pancreatic release of proinflammatory cytokines (IL-1*β* and IL-6) [90].

### 4.2. Baicalin

Baicalin is a flavone glucuronide known and used as an anti-inflammatory agent [140]. It is extracted from* Scutellaria* spp. (skullcap), fam. Lamiaceae (particularly from* S. baicalensis*) and from* Erigeron breviscapus*, fam. Asteraceae [140, 141].

In the intestine, baicalin is hydrolysed to baicalein (the aglycone form) by *β*-glucuronidase, this conversion being required for absorption. Afterwards, baicalin is regenerated, by reglucuronidation, in the liver and intestine [142, 143].

#### 4.2.1. *In Vitro* Studies

Studies performed on macrophagic cells demonstrated the anti-inflammatory and antioxidative effects of baicalin [140] which allows us to surmise a favourable effect in diseases characterized by systemic inflammation, such as AP, especially SAP.

*Inflammation*. Baicalin blocked the activation of the macrophages and lipopolysaccharide (LPS) induced synthesis of proinflammatory mediators [tumor necrosis factor *α* (TNF-*α*), endothelin-1 (ET-1), and thromboxane A2 (TXA2)] in RAW264.7 (macrophage-type) cells [140].

*Oxidative/Nitrosative Stress*. Baicalin staved off the augmentation in nitric oxide (NO) production and inducible nitric oxide synthase (iNOS) expression promoted by LPS and Interferon-*γ* (IFN-*γ*) although it did not directly alter the activity of iNOS in RAW264.7 cells and pMΦs. It also hindered the generation of reactive oxidative species (ROS) while increasing the intracellular level of superoxide dismutase (SOD) [140].

#### 4.2.2. Animal Studies

Baicalin is able to take the edge off AP by a combination of dampened inflammation and oxidative stress and heightened apoptosis, an effect extending beyond the pancreas, to the other organs suffering under the systemic impact of AP, including the main tracts of the organism [digestive (liver, intestinal mucosa), respiratory (lung), and urinary (kidney)] and the lymphatic organs (spleen, thymus), which results in milder systemic disease and improved survival [91, 93, 94].

*Apoptosis*. Baicalin promotes apoptosis (higher apoptosis index) in the pancreas (and also in the intestinal mucosa, lymph nodes, and spleen [93]) by activating caspase-3 [92] and by increasing the expression of Bax protein [91] not only in the pancreas but also in the lung and the intestinal mucosa (however it decreases it in spleen and lymph nodes) [93]. The apoptosis-inducing effect is at work also in the kidney (higher renal apoptotic indexes [91]), at least partially due to a decrease in the renal Bcl-2 protein (although the level of Bax protein was not influenced) [91].

*Necrosis*. Baicalin diminishes amylase [44] and the pathological severity scores in pancreas and multiple other organs [94] and is at least as efficient as the currently used drugs (somatostatin/octreotide) in the treatment of AP [92].

*Systemic Effects*. Baicalin decreases the systemic severity of AP, as proven by lower endotoxinemia and improved survival [91, 93, 94], while relieving the noxious effects on the kidney (where it preserves the normal histology and function: less severe renal pathological changes [91] and lower serum BUN [91]) and on other organs (it diminishes the pathological changes in multiple organs, including pancreas [44, 93], lung [44, 93], ileum, and lymph nodes [93]), which points to a putative ability to prevent the development of multiorgan dysfunction syndrome.

*Inflammation*. Baicalin decreases the inflammatory response, as indicated by lower markers of inflammation (IL-6 [44, 91], TNF-*α* [44, 91–93]) and of leukocyte recruitment (P-selectin, also involved in the aggregation of platelets) [92, 94] in rats with SAP. The pathways by which this effect is achieved pass through the downregulation of activators such as ET-1 [91, 94], PLA2 [91, 94], and TLR4 [44].

*Oxidative/Nitrosative Stress*. Baicalin decreases nitrosative stress, translated in lower levels of NOin rats with SAP [91, 94].

### 4.3. Crambene

Crambene is an unsaturated nitrile (1-cyano-2-hydroxy-3-butene) derived from the breakdown of (epi)progoitrin glucosinolates found in many cruciferous plants (e.g., brussels sprouts) [144, 145]. It was initially considered to be a selective pancreatotoxin (when administered at high doses, e.g., 100–200 mg/kg body weight/day), due to its capacity to induce changes consistent with apoptosis of pancreatic acinar cells, infiltration of pancreatic lobules by macrophages and acinar atrophy [146–148]. Unexpectedly, its toxicity was associated with a significant and persistent increase in the pancreatic glutathione (GSH) [149]. After more research was performed, its proapoptotic activity, when adequately manipulated by dosing and choosing the route of administration, turned to be a beneficial one, useful in the mitigation of acute pancreatitis in experimental studies. For instance, moderate oral doses 30–100 mg/kg increased pancreatic GSH levels without any pancreatotoxicity, while a single 50 mg/kg intravenous dose induced apoptosis, and 100 mg/kg caused severe pancreatotoxicity with necrosis in male Fischer 344 rats [150].

#### 4.3.1. *In Vitro* Studies

*Apoptosis*. Treatment with 2 mM crambene for 3 h induced apoptosis of isolated pancreatic acinar cells (confirmed by increase of caspase-3, caspase-8, and caspase-9 activities), but not necrosis [151]. The phytocompound induced the collapse of mitochondrial membrane potential, followed by cytochrome c release from the mitochondria, but neither TNF-alpha nor Fas ligand production by pancreatic acinar cells was changed. These results suggest the involvement of the intrinsic pathway of apoptosis (caspase-3, caspase-9) but do not exclude the simultaneous activation of extrinsic pathway (caspase-9) [151]. A coculture study revealed that CD36-positive macrophages might play an important role in phagocytosis of apoptotic acinar cells induced by 2 mM crambene treatment, and phagocytosis seems to suppress the inflammatory response by increasing the release of anti-inflammatory cytokine IL-10 [95].

#### 4.3.2. Animal Studies

*Apoptosis/Necrosis*. Pretreatment with crambene induced apoptosis of pancreatic acinar cells and reduced the extent of cerulein-induced necrosis, edema, and hyperamylasemia [51]. The reduction of pancreatitis severity of pancreatitis is maximal when crambene was administered 12 hrs before cerulein administration [51].

*Inflammation*. Another study revealed the mechanism through which apoptosis reduces the severity of acute pancreatitis: clearance of apoptotic acinar cells by CD36-positive macrophage induced the release of anti-inflammatory cytokines IL-10 and TGF*β*1 [95].

### 4.4. Curcumin

Curcumin, a polyphenol (a curcuminoid, i.e., a linear diarylheptanoid), is the primary active constituent of turmeric* (Curcuma longa)* and other related species. It is poorly absorbed and the serum concentration declines rapidly, and therefore curcumin has limited systemic bioavailability [152, 153]. Metabolism of curcumin involves biotransformation to dihydrocurcumin, tetrahydrocurcumin, and conversion into monoglucuronide and sulphate conjugates, which were detected in plasma of human subjects [154, 155]. Taking into account the reduced absorption and rapid plasma clearance of curcumin, the scientists looked for solutions to improve its systemic bioavailability. Piperine was found to be an effective bioenhancer for curcumin by increasing its absorption and by inhibiting its hepatic and intestinal glucuronidation [156].

Clinical studies showed that, even at high doses (such as to 12 g daily for several months), curcumin therapy is devoid of side effects but for mild nausea and diarrhea [153, 157].

#### 4.4.1. Animal Studies

There are a few studies confirming the beneficial effect of curcumin in AP by improving the balance between apoptosis and necrosis-inducing processes. Several animal models of pancreatitis have been used, both ethanol-dependent and non-ethanol-dependent, employed as inductor agents: arginine [96], sodium taurocholate [97, 98], cerulein [99, 100], and low-dose CCK-8 in previously ethanol-sensitized animals [100].

*Apoptosis*. Curcumin promotes apoptosis by activating caspase-3 [96, 98].

*Necrosis*. Curcumin reduces the pancreatic injury [99], as reflected by improved histopathologic scores [98] and lower levels/activities of pancreatic enzymes/amylase [96, 97, 99], lipase [96], and trypsin [98].

*Systemic Effects*. Curcumin also ameliorates the deleterious effects of pancreatitis on other organs and territories, as indicated by a decrease in transaminases levels [99], in the ascites volume [97], and in the airway hyperreactivity [101]. The latter was studied on a model of ischemia/reperfusion-induced pancreatic injury and the benefic effect of curcumin appears to be mediated by diminished expression of iNOS and TNF-*α* in the lung tissue [101].

*Inflammation*. The appeasing effect of curcumin on inflammation was pointed out by lower levels of the inflammatory cells (leukocytes [101]) and mediators such as cytokines (IL-6 [97, 98, 100], TNF-*α* [96–101]) and chemokines [98], including KC [100] and ENA-78 [96]. At least some of the explanation lies with the depressing effect on the activators of inflammation, specifically on the transcription factors (AP-1 [98], NF-*κ*B [97, 98, 100], and NF-*κ*B-p65 [99]), presumably by means of decreasing the activators of transcription factors (TLR4 [97]) and increasing the inhibitors of transcription factors (PPAR*γ* [99]).

*Oxidative/Nitrosative Stress*. Curcumin decreases the activity of the enzymes responsible for the oxidative aggression (MPO [96], iNOS [98, 100, 101]), which translates into lower levels of the products of oxidative aggression (HO^*∙*^ [101], NO [98, 101], and protein carbonyls [96]).

#### 4.4.2. Human Clinical Studies

*Oxidative Stress*. A single-blind, randomized, placebo-controlled study evaluated the effects of oral mixture of curcumin (500 mg) with piperine (5 mg) for 6 weeks on the clinical evolution and oxidative stress biomarkers in 20 patients with tropical pancreatitis, a type of chronic pancreatitis. The herbal formulation significantly reduced the erythrocyte malondialdehyde (MDA) level, a marker of lipid peroxidation, and increased the GSH level, without influencing the pain [158]. Whether curcumin would be beneficial or not in acute pancreatitis in humans remains to be investigated.

### 4.5. Embelin

Embelin is a benzoquinone derivative (2,5-dihydroxy-3-undecyl-1,4-benzoquinone) identified in marlberry (*Ardisia japonica*, fam. Primulaceae) and various* Embelia* species such as Indian* Embelia* or vidanga (*Embelia ribes* Burm, fam. Primulaceae) and African* Embelia* (*Embelia schimperi* Vatke, fam. Primulaceae) [159–162].

#### 4.5.1. *In Vitro* Studies

*Apoptosis*. Embelin is known as a cell-permeable, small-molecular weight, and potent inhibitor of XIAP, due to its capacity to bind to the Baculovirus Inhibitor of apoptosis protein Repeat 3 (BIR3) domain in XIAP (the binding site for caspase-9) [160]. Thus, by preventing XIAP interaction with caspase-9, it allowed the activation of initiator caspase-9 in prostate cancer cells, which led to apoptosis. Embelin showed dose-dependent proapoptotic activity in pancreatic cancer cells also, and the effect was potentiated in combination with ellagic acid [163]. In another study, embelin inhibited XIAP expression in adenoviral vector encoding human X-linked inhibitor of apoptosis transduced human pancreatic islets [164]. The inhibition was even to lower level than the basal XIAP expression in normal islets. Pancreatic stellate cells represent another target of embelin (10 and 20 *μ*M), their apoptosis being significantly increased by the phytocompound (by 3- and 6-fold, resp.)* in vitro* [163]. The proliferation rate of PSC was also increased, when compared to control [163].

*Inflammation.* Embelin inhibited the biosynthesis of eicosanoids in human polymorphonuclear leukocytes and monocytes in a potent, selective, noncompetitive, and reversible way, by directly targeting the human 5-lipoxygenase (5-LO) and microsomal prostaglandin (PG) E_2_ synthase (mPGES)-1 [165]. Therefore embelin was proposed as a novel chemotype for designing dual 5-LO/mPGES-1 inhibitors [165]. This inhibition did not correlate with the antioxidant properties of embelin. On the other way, human 12-LO, 15-LO, COX-1, COX-2, and cytosolic phospholipase A_2_ were not significantly affected by 10 *μ*M embelin [165].

#### 4.5.2. Animal Studies

*Apoptosis versus Necrosis.* In mouse cerulein pancreatitis, subcutaneous injection of embelin (20 mg/kg) for 5 consecutive days significantly increased the activity level of caspase-9 (direct way), as well as caspase-3 and caspase-8 (indirect way), which were correlated with a 3-fold increase in apoptosis [7]. These changes were associated with a decrease in necrosis and normalization of pancreatic histology, leading to the conclusion that embelin, in addition to its proapoptotic activity, displays antinecrotic activity, modulating the balance between apoptosis and necrosis in acute pancreatitis [7].

### 4.6. Emodin

Emodin is an anthraquinone derivative (1,3,8-trihydroxy-6-methyl-anthraquinone) found in rhubarb (*Rheum* spp., fam. Polygonaceae), buckthorn (*Rhamnus* spp., fam. Rhamnaceae), and Japanese knotweed (*Fallopia japonica*, fam. Polygonaceae) with proven anticancer and anti-inflammatory effects [166]. Emodin has been used clinically for the treatment of AP for many years in China [167]. It is worth mentioning that a meta-analysis on the prospective randomized controlled studies using Chinese herbal medicine in nonbiliogenic severe AP (SAP) revealed rhubarb as the most often used (in 19 out of 22 studies) botanical drug [168].

#### 4.6.1. *In Vitro* Studies

*Apoptosis versus Necrosis.* Emodin decreased the severity of AP (as demonstrated by lower amylase levels) by turning the acinar cells from the destructive pathway of necrosis to the sounder one leading to apoptosis, by means of reducing the calcium overload in the cytoplasm. The underlying mechanism seems to rely at least partially on the downregulation of the ER stress response in association with diminished expression of some of the involved proteins acting as ER stress transducers (as part of the unfolded protein response): Bip (ER chaperone immunoglobulin-binding protein), PERK (protein kinase-like ER kinase), ATF6 (activation transcription factor 6), and IRE1 (inositol-requiring protein 1) [113].

*Inflammation*. Emodin inhibited TNF-*α*-induced NF-*κ*B activation and adhesion molecule expression (e.g., ICAM-1, VCAM-1, and ELAM-1) in human umbilical vein endothelial cells [169]. A study employing rat pMΦs demonstrated the ability of emodin to block a purinergic receptor P2X_7_R and thus to antagonize the ability of ATP to stimulate IL-1*β* release and to hinder phagocytosis [170]. These inflammation-hampering effects explain the capacity of emodin to counteract the local and systemic effects of inflammation-generating diseases such as pancreatitis [167].

*Oxidative Stress*. A study done on rat pMΦs highlighted emodin's suppressive action on ATP-triggered ROS production [170].

#### 4.6.2. Animal Studies

*Blood Flow*. The onset of SAP is accompanied by a drop in the pancreatic blood flow which is at least partially the consequence of surging thromboxane B2 (TXB2) and plunging 6-keto-PGF_1a_ and PGE_2_ levels. Emodin reversed the ischemia typical for the early stages of SAP [102] while (and supposedly by means of) restoring the balance of vasoconstrictor/vasodilator eicosanoids [102, 103].

*Necrosis*. Emodin attenuated the pancreatic injury (lower amylase [44, 103, 108, 111, 114, 115] and lipase [103] and milder pathological changes/histological score [44, 103, 104, 108, 114, 167], including edema [103, 104, 108, 115], extravasation [104], vacuolization [108], hemorrhage [103, 115], and inflammatory infiltration and necrosis [103, 108, 115]) an effect augmented by concomitant early enteral nutrition (EEN) [115] or baicalin treatment [44]. Fastening the epithelial and endothelial barriers in the pancreas [104] and in the lungs [105] by increased expression of some of the proteins involved in the intercellular tight junctions (claudin-4 [105] and claudin-5 and occludin [104, 105]) may contribute to antiedematous effect by diminishing paracellular permeability [104, 105]. 

*Systemic Effects*. On animal models of AP, emodin prevented multiorgan failure [167], reduced the general severity of the disease (diminished mortality [103, 114, 115] and endotoxin and lactate levels [115]) as well as the impact on other organs/territories {*peritoneum* (less ascites [103, 114, 115]),* liver* (lower ALT and AST [115], improved histological score [167]),* lungs* (milder pathological changes of acute lung injury (ALI) [44, 104, 111, 167]), and* bowel* (less damage [167] correlated with better intestinal transit and lower inflammatory activators and mediators (NF-*κ*B-p65, TNF-*α*, IL-1*β*) [107], ameliorated intestinal flora [102], and improved survival and barrier function of intestinal mucosal cells [167] resulting in diminished passage of bacteria and endotoxin [112])}, an effect boosted by the association with EEN [115] or with baicalein/baicalin in the case of ascites [114] and pulmonary injury [44]. The protective effect against ALI (translated in less pulmonary edema and inflammation) seems to be associated with the increased lung expression of aquaporins (AQP) 1 and 5 (AQP1 in the alveolar capillary endothelial cells and AQP5 in the alveolar type I and II cells), transmembrane proteins dedicated to water transport, whose decrease is strongly coupled with the development of ALI and pulmonary edema) [111].

*Inflammation*. Emodin decreased the level of inflammatory markers/mediators (TNF-*α* [44, 104, 108, 111, 114, 115], IL-6 [44, 104, 108, 114], IL-1*β* [108], and CRP [114, 115]), an effect magnified by concurrent EEN [115] or baicalin/baicalein administration in the case of TNF-*α* [44, 114]. While exploring the possible pathways leading to this inflammation shut down, emodin has been proven to block the upregulation of TLR4 (even more so if associated with baicalin) [44], the activation of NF-*κ*B [108], and the expression of P-selectin [167]. Emodin has also been shown to hasten the resolution of inflammation by augmenting the expression of membrane-bound cluster of differentiation 14 protein and of intercellular adhesion molecule-3 in pMΦs, which may promote the phagocytosis of apoptotic neutrophils, thereby encouraging inflammation fade-out [109].

*Oxidative Stress*. Emodin decreased the activity of hepatic and pancreatic MPO [115] and lowered MDA [108, 115] (an effect amplified by simultaneous EEN [115]) and increased the activity of SOD [108].

#### 4.6.3. Human Clinical Studies

Although emodin pancreatoprotective activity was not evaluated as an isolated compound in human clinical studies, there are already available results on the effects of emodin containing medicinal plants in patients with pancreatitis.

*Inflammation, Systemic Effects*. In a study done on 126 patients with severe AP comparing rhubarb-assisted EEN with EEN alone and with parenteral nutrition (followed by enteral nutrition after a fortnight's delay), rhubarb-assisted EEN accelerated symptom resolution (abdominal pain, transit disorders, and fever) and recovery, decreased the severity of the disease (APACHE score) and of the systemic inflammation (white blood cell count, CRP, and IL-6), and reversed hepatic and renal injury [171].

In other studies, scientists have evaluated the effects of rhubarb administrated via different ways in subjects with SAP (e.g., acute hemorrhagic-necrotic pancreatitis) and found positive effects on the inflammatory markers [172, 173].

### 4.7. Flavanones Hesperidin and Naringin

Hesperidin is a flavanone glycoside (the aglycone is hesperetin) found primarily in citrus fruits [174] but also in the Chinese herbal formulation Da-Cheng-Qi decoction (DCDQ) [117], known especially for its capillary-wall-strengthening, antioxidant, anti-inflammatory, anticarcinogenic, and antiallergic effects [174].

Naringin is a flavanone glycoside (the aglycone is naringenin) found primarily in tomatoes and various citrus fruits (especially in grapefruit, accounting for its bitter taste) with proven antioxidant, anti-inflammatory, antiapoptotic, and anticancer/chemopreventive properties, efficient in various models of cardiovascular, neurodegenerative, metabolic (diabetes mellitus), rheumatological (including osteoporosis), and oncological disorders [175].

#### 4.7.1. *In Vitro* Studies

*Apoptosis*. Hesperidin is one of the compounds imparting healing proficiency in AP to the Chinese herbal formulation DCQD, augmenting the cellular survival and apoptosis-to-necrosis ratio and dampening the ROS generation [117].

#### 4.7.2. Animal Studies

*Inflammation and Oxidative/Nitrosative Stress*. In an experiment done on an animal model with cerulein-provoked AP, hesperidin reduced the severity of the disease (amylase level) and the intensity of the inflammatory process (edema, leukocyte infiltration) and of the ROS and NO generation (as measured by chemiluminescence using luminol and lucigenin) [116].

### 4.8. Lignans (Magnolol, Honokiol, and Nordihydroguaiaretic Acid)

Nordihydroguaiaretic acid (NDGA) is a lignan extracted from creosote bush,* Larrea tridentata* (Zygophyllaceae), with documented efficiency in cancer prevention, diabetes, infections, and fertility regulation [176].

Magnolol and honokiol are lignans extracted from* Magnolia officinalis* (Magnoliaceae) able to fend off oxidative aggression, inflammation, cancer, and infections [177].

#### 4.8.1. Animal Studies

*Apoptosis*. NDGA prevents acinar cells necrosis by enhancing Bcl-2 expression and promotes apoptosis (as confirmed by the increased number of apoptotic cells) by encouraging the phosphorylation of PP2A and the conversion of procaspase-3 in caspase-3. NDGA seems to impact both sides of apoptosis/necrosis balance (furthering the first and encumbering the second) by its influence on the histone H3 modifications, resulting in altered expression of the genes involved in inflammatory/apoptotic cascade [122].

Magnolol [117] and honokiol [118] are two of the compounds in DCQD that have shown apoptosis-enhancing properties.

#### 4.8.2. Necrosis/Injury

NDGA reduced the injury to the pancreatic tissue, with resultant milder edema (reflected by the increase in pancreas weight), histological damage, and plasma amylase surge [122].

NDGA augmented the expression of several heat shock proteins (DNAJ C15, HSPD1, and HSP 27), whose cytoprotective effect helped hindering the development of AP [122].

Magnolol reduced the LDH release from the pancreatic cells [117], the seric level of amylase, and the severity of pancreatic histopathologic alteration [121]. Honokiol also showed an ability to prevent pancreatic cells demise, reflected by lower LDH release [118].

*Inflammation*. NDGA attenuated the inflammation (and its consequence, necrosis) in the pancreatic tissue (lower levels of MPO, a marker of leukocyte infiltration, and of TNF-*α*) by blocking the NF-*κ*B pathway (both decreased expression and activation by p38 phosphorylation) [122].

Magnolol quenches the inflammatory response in AP by reverting the imbalance between the two involved types of T helper (Th) cells, Th1 and Th2, a high Th1/Th2 promoting inflammation. The dendritic cell- (DC-) directed preferential differentiation of Th0 to Th1 (rather than to Th2) drives the inflammation in AP, but magnolol was able to increase the myeloid-to-lymphoid DC ratio, as well as the IL-10/IFN-*γ* ratio, thereby switching the Th0 differentiation from the Th1 to Th2 and decreasing the Th1/Th2 ratio from the high proinflammatory levels typical for AP to some lower values more congenial with an appeased local and systemic inflammatory process [121].

*Oxidative Stress*. NDGA lowered the level of ROS (thiobarbituric acid reactive substrate) and strengthened the antioxidative mechanisms (increased SOD and GSH) [122].

### 4.9. Ligustrazine

Ligustrazine (tetramethylpyrazine) is a phytochemical isolated from Chinese herb* chuānxiōng* (*Ligusticum wallichii*, fam. Umbelliferae), used in Traditional Chinese Medicine (TCM) for more than 2000 years for invigorating blood and moving stagnant Qi or blood [178, 179]. Pancreatitis is not listed as a common indication of the plant, which is mainly used for gynaecological problems and headaches [179]. Nevertheless, the activation of blood circulation to dissipated blood stasis (one of the plant ethnopharmacological activities) is part of TCM strategy in pancreatitis therapy [180].

#### 4.9.1. Animal Studies

*Apoptosis versus Necrosis*. Ligustrazine induced pancreatic acinar cell apoptosis in rats with SAP [119, 181], accelerating the process especially at an early stage of the disease [107]. Interestingly, although ligustrazine promoted apoptosis of acinar cells, it prevented cell apoptosis in the liver and kidney [181]. In another study, the phytocompound alleviated necrosis in rats with taurocholate-induced acute hemorrhagic necrotizing pancreatitis [120].

*Inflammation.* Ligustrazine reduced the levels of TNF-*α*, IL-1*β*, IL-6, amylase, pancreatic MPO activity, and the degree of inflammatory cell infiltration in pancreas in rat models of AP [119, 182]. This phytocompound seemed to alleviate the inflammatory complications of pancreatitis due to its capacity to reduce pathological changes in the lung, stomach, small intestine, kidney, and immune organs (thymus, spleen) [183–186]. In one of these studies, ligustrazine was more effective than the other two phytochemicals (kakonein and Panax notoginsenosides) in alleviating the tissue damage in the small intestine and immune organs of SAP rats [183], but less effective in protecting pancreas than Panax notoginsenosides, although its action was more comprehensive [181].

*Oxidative Stress*. Ligustrazine exerted ROS scavenging properties and decreased the serum level of lipid peroxides in rats with acute hemorrhagic necrotizing pancreatitis [120].

### 4.10. Resveratrol

Resveratrol (trans-3,5,4′-trihydroxystilbene) found in various plants such as grapes, blueberries, peanuts, pistachios, soy, and giant knotweed* (Polygonum cuspidatum)* beans showed in various experimental models antiapoptotic, anti-inflammatory, and antioxidant features; therefore it is a potential candidate for pancreatitis treatment [187–190]. It is metabolised by colonic microbiota and converted into dihydroresveratrol, when orally administered [191]. Dihydroresveratrol is also produced by certain plant species (e.g., Orchidaceae family,* Cannabis sativa*) as a phytoalexin against stressors [192, 193].

#### 4.10.1. *In Vitro* Studies

*Apoptosis.* Resveratrol is a blocker of pancreatic sulfonylurea receptors 1 SUR1/KIR6.2 ATP-sensitive K channels [194]. Sulfonylureas are reported to have an apoptotic activity in cultured human islets, through a mechanism involving blockade of K^+^ ATP channels and depolarization-induced Ca^2+^ influx into the cell [195–197]. Correlated with this SUR1 ligand activity, the phytocompound displayed a proapoptotic effect, stronger than that of glibenclamide, in SUR1-expressing recombinant human embryonic kidney (HEK) cells, but also in native *β*-cells. Apoptotic parameters such as cell detachment, caspase-3, caspase-9, and caspase-12 activities and degree of nuclear fragmentation were enhanced after resveratrol treatment. The effect appeared after incubation with 100 *μ*M resveratrol for at least 24 h [194].

#### 4.10.2. Animal Studies

*Apoptosis versus Necrosis*. Significant therapeutic effect of resveratrol was noticed in rats with AP by inducing apoptosis of pancreatic acinar cell as a consequence of upregulated FasL gene expression [198]. Resveratrol and dihydroresveratrol protect animals against pancreatitis complications (e.g., lung damage, intestinal barrier alteration, and brain and hepatic injury) [126, 127, 130, 199]. Scientists claim that the mechanism responsible for this protective activity is based on the upregulation of antiapoptotic Bcl-2 and downregulation of proapoptotic Bax and caspase-3 [127, 199]. It seems that resveratrol, like ligustrazine, has a selective bioactivity, being proapoptotic on acinar cells and antiapoptotic on other types of cells (e.g., hepatocytes).

*Inflammation.* Various scientists noticed that resveratrol and its metabolite (dihydroresveratrol) exerted a dose-dependent anti-inflammatory effect through NF-*κ*B inhibition and decreased expression of TNF-alpha, IL-1*β*, IL-6, and IL-8 in the pancreas, pMΦs, and other target organs (e.g., lungs), in various animal models of pancreatitis (e.g., sodium taurocholate, cerulein, and CCK-8-induced disease) [123–125, 129]. There are contradictory findings concerning the effects of resveratrol on the production of NO and NOS: activated endothelial NOS was found to be protective against cerulein-induced AP in mice, while inducible NOS exhibited proinflammatory effects in the same animal model [200–202].

*Oxidative Stress.* Resveratrol treatment induced an increase in the pancreatic SOD and a decrease of MDA within the first 6 hours of SAP induction in SD rats [128]. Dihydroresveratrol ameliorated the pancreatic oxidative damage by inhibiting the activities of NADPH oxidase and MPO and by restoring the glutathione pool [191].

#### 4.10.3. Human Clinical Studies

A multicenter, prospective, randomized controlled clinical trial, registered on https://www.clinicaltrials.org/ in 2016 (ClinicalTrials.gov Identifier: NCT02947932), is currently running in order to evaluate the efficacy of resveratrol in preventing post-Endoscopic Retrograde Cholangiopancreatography (ERCP) pancreatitis [203].

### 4.11. Rhein

Rhein is an anthraquinone derivative (endowed with a carboxyl group, hence alternative names of cassic or rheic acid) found in* Rheum* spp. (rhubarb) (like* R. undulatum*,* R. palmatum*, etc.) and in* Senna* spp. (*S. reticulata*,* S. alexandrina,* etc.). It is also a component of the Chinese herbal medicine Da-Cheng-Qi decoction [53].

#### 4.11.1. *In Vitro* Studies

*Apoptosis*. Rhein promoted apoptosis in rat pancreatic acinar cells, dose-dependently enhancing the ratio of apoptotic-to-necrotic cells, as well as the level of p53, cytochrome C, and caspase-3, and Bax/Bcl-2 ratio, which indicate the ability to channel the injured cells to die by mitochondrial apoptosis rather than by necrosis [53].

DCQD is of proven benefit in AP [204] and in its complications [205–208]—a search for the biochemical substrate thereof revealed four compounds (rhein, magnolol, hesperidin, and naringin) as champions, with rhein in the leading position. Pretreated with any of the four compounds, cultured pancreatic cells exposed to the deleterious effects of cerulein were healthier in life and more responsible in death (better viability, higher apoptosis, lower necrosis, and diminished ROS production). Among the four, however, rhein was the best not only in pharmacodynamic terms but also in pharmacokinetic characteristics: despite having the lowest concentration in DCQD, it reaches a much higher plasmatic level than the other three (the study was performed on a rat model of sodium taurocholate-induced AP) [117]. Some of the other components of DCDQ, particularly naringin (a flavanone) and honokiol (a lignan), seem to act synergistically with rhein, the combination of three being more efficient in spurring apoptosis compared to each of the individual components alone and compared to each combination of two components [118].

*Apoptosis*. Pretreatment with rhein protected the pancreatic cells from the cerulein-induce injury as proved by the drop in LDH release [117].

#### 4.11.2. Animal Studies

*Inflammation and Systemic Effects*. The usefulness of rhein in the treatment of AP and its complications is severely limited by its failure to accumulate in the pancreas and other territories (such as lung), which may be nevertheless overcome by coupling it with a derivative of propane diamine which increments its affinity for pancreatic and lung tissue. Given to animals subjected to experimental AP, this conjugated compound (HPDM-rhein) reduced the severity of AP (lower amylase, milder injury to the acinar cells in terms of vacuolization and destruction), the neutrophilic inflammatory infiltrate (indicated by MPO and histological data), the local and systemic inflammatory reaction (lower levels of IL-6 and TNF-*α* in plasma, pancreas, and lung), the lung histological damage (alveolar wall thickening by edema and cellular infiltration), and the volume of ascites. Interestingly, many of these actions are also displayed by the unconjugated rhein, but at a much lower intensity, as not to differ significantly from the untreated controls, the only exception being IL-6 at 3 hours [131].

### 4.12. Rutin

Rutin is a rhamnoglucoside of quercetin found in high amounts in several citrus fruits, grapes, black tea, apple skin peels, and amalaki fruits* (Emblica officinalis)*. Rutin was found to be less absorbed than other quercetin glucosides (the bioavailability of rutin was ~80% less than that of quercetin glucosides) [209, 210]. This low bioavailability is correlated with rutin conversion by gut microflora in the large intestine to various compounds (e.g., 3,4-dihydroxyphenylacetic acid, 3,4-dihydroxytoluene) [211, 212].

#### 4.12.1. *In Vitro* Studies

*Inflammation.* 3,4-Dihydroxytoluene, a metabolite of rutin, exerted anti-inflammatory effects in LPS stimulated RAW 264.7 macrophages by deactivating NF-*κ*B signalling [212]. Therefore this metabolite may be a potential adjuvant remedy against local and systemic inflammation in pancreatitis.

#### 4.12.2. Animal Studies

*Apoptosis versus Necrosis*. In L-arginine induced AP model rutin decreased the pancreatic injury (less necrosis, edema and infiltration, and lower serum levels of the pancreatic enzymes) but also furthered apoptosis (enlarging the population of apoptotic cells in the pancreas) [132]. Paradoxically, rutin could also antagonize factors involved in other types of programmed cell death, such as pyroptosis. For instance, rutin treatment downregulated the expression of caspase-1 and pyrin domain (PYD) of the apoptosis-associated speck-like protein (ASC), while it upregulated the expression of CARD (caspase activation recruitment domain (CARD) in male Wistar rats with cerulein-induced pancreatitis and fed on ethanol [133]. This dual effect of rutin on programmed cell death may be explained as being dependent on the type of mechanism involved and the degree of inflammation: proapoptotic activity during the early phase of pancreatitis and inflammation (first few days) [132], and antipyroptotic activity during the chronic stage of pancreatitis and inflammation (during the following weeks) [133].

*Inflammation.* Rutin tempered inflammation (lower CRP) and protected pancreas against inflammatory injury in both acute and chronic pancreatitis models via several mechanisms: decreased expression of cytokines (IL-1*β*, IL-6, and TNF-*α*) and decreased neutrophil infiltration (measured as MPO activity) [24, 132–134]. Rutin supplementation also preserved pancreatic microarchitecture, improved food consumption, and maintained net weight gain in rats with alcohol-induced and high-fat-induced chronic pancreatitis [24].

*Oxidative Stress.* In L-arginine induced AP model, beside alleviating abdominal hyperalgesia, rutin also diminished the oxidative stress (reflected in the ameliorated 3-nitrotyrosine level) and hindered lipid peroxidation (by decreasing MPO), while boosting SOD and catalase [132].

## 5. Discussions

### 5.1. Multitargeting: One Phytochemical-More Targets

Most of the phytochemicals are highly pleiotropic molecules with multiple targets and mechanisms of action (Table 3) [213]. All the phytochemicals selected, beyond their anti-inflammatory and antioxidant activity, displayed the potential to directly regulate the balance between apoptosis and necrosis of acinar cells, either by enhancing apoptosis or by decreasing necrosis. They multitarget the crossroads between apoptosis and necrosis pathways, which represent turnpoints in the progression of inflammatory changes in pancreatitis.

We must mention here certain recent efforts in application of the network pharmacology for selection of the best multitarget active phytochemicals as potential AP innovative drugs. Resveratrol was identified as the phytocompound with the highest number of potential targets (degree = 38), followed by emodin (35), curcumin (32), honokiol (16), baicalin (15), and ligustrazine (13) [216].

### 5.2. Synergism: More Phytochemicals-One Target

We also highly value the studies on phytoceuticals synergy in AP [118], although they are unusually rare. Evaluation of traditional polyherbal formulation used for millenia in TCM, Ayurveda, or other ethnomedical systems may lead to the discovery of precious phytosynergisms with high therapeutic efficacy in AP.

### 5.3. Selective Bioactivity

A peculiar feature of certain phytochemicals is represented by their selective bioactivity (e.g., ligustrazine—proapoptotic on acinar cells, but antiapoptotic on liver and renal cells) [181], which may represent an advantage over the synthetic drugs.

### 5.4. Limitations of Our Hypothesis on Proapoptotic Dependent Efficacy of Phytoceuticals in AP

By contrast to these phytocompounds that promote apoptosis, there are others that block apoptosis and still favourably influence the evolution of AP (by attenuating inflammation, necrosis, and oxidative stress in the pancreatic tissue and by preventing its local and systemic complications): daphnetin (a coumarin from* Dracaena marginata*) [217], green tea polyphenols [218], and proanthocyanidins [214]. We could estimate that the rate and the degree of apoptosis, which directly influence the amount of proinflammatory DAMPs released, might explain this paradox: proapoptotic phytoceuticals exhibit benefits when apoptosis rate is extremely low, while antiapoptotic phytoceuticals display positive effects when apoptosis is exacerbated, and DAMPs phagocyting cells are overwhelmed. Nevertheless, it is desirable to perform comparative studies on the efficacy of pro- and antiapoptotic phytocompounds in AP, in order to reveal their hidden mechanisms of action and all their possible interferences with apoptosis and phagocytosis pathways in AP.

### 5.5. Research Challenges

Despite their advantages, the low bioavailability of certain phytoceuticals represents a serious limitation. Several mechanisms are claimed to be responsible for this pharmacokinetic behaviour: rapid sulphate conjugation by the intestine/liver (e.g., resveratrol) or low solubility (e.g., curcumin) [219, 220], but potential solutions were already proposed (e.g., use of bioenhancers, such as piperine) [156].

Future research challenges are represented by the evaluation of the phytochemical bioactivity in large-population clinical studies and identification of the potential side effects or interactions with synthetic drugs. It is also worth investigating the combined administration of several phytochemicals or of phytochemicals together with synthetic drugs to determine the optimal mixture for cost-effective therapies. Development of food items fortified with phytochemicals with clinically proven pancreatoprotective or pancreatoregenerative properties would contribute to the implementation of this theoretical knowledge into practice.

## 6. Conclusions

Activation of apoptosis via various mechanisms and/or inhibition of necrosis seems to represent the essential key for decreasing the severity of the acute pancreatitis. All the phytochemicals selected, belonging to such diverse classes as polyphenols (curcumin, resveratrol), flavonoids (baicalin, hesperidin, naringin, and rutin), lignans (honokiol, magnolol, and nordihydroguaiaretic acid), anthraquinones (emodin, rhein), sesquiterpene lactones (artemisinin), nitriles (crambene), and alkaloids (ligustrazine), target the balance between apoptosis and necrosis. Apart from tipping the apoptosis/necrosis balance, the phytochemicals displayed other specific protective activities: inhibition of inflammasome (e.g., rutin), suppression of neutrophil infiltration (e.g., ligustrazine, resveratrol), and antioxidant activity. These phytoceuticals may represent a promising complementary therapy for acute pancreatitis, having also few rare adverse reactions, and sometimes even better results than the standard treatment [92]. Nevertheless, there is a shortage of human evidence, and further studies are required to provide solid basis to justify their use in the treatment of this disease.

## Figures and Tables

**Table 1:** Animal models of pancreatitis.

Chemical inducer	Characteristics	Pancreatitis type	References
Bile salts	Mechanical temporary blockage of bile duct, detergent effect of the bile salts, and hemorrhagic necrosis	Severe AP	[16]

Cerulein	Stimulation of pancreatic enzyme production, inhibition of zymogen granules exocytosis, NADPH oxidase activation, increased reactive oxygen species generation, NF-*κ*B activation, cytokine expression, and acinar cells death	Mild AP CP (repeated exposure)	[17–19]

L-arginine	Increased production of amylase, lipase, and trypsinogen, markedly swollen mitochondria, and degenerative changes of intracellular organelles and nuclei	Severe necrotizing AP CP	[20, 21]

Alcohol +/− high fat diet	Stellate cell activation, fibrosis, and acinar cell mass shrinkage	Mild CP	[22–24]

Dibutyltin dichloride	Edema (24 h), mononuclear cells infiltration (day 7), bile duct epithelium necrosis, upregulation of transforming growth factor-*β*1, and fibrosis with increased collagen type I production	AP CP	[25]

CCK	Increased plasma amylase, lipase, trypsin-like immunoreactivity, pancreatic parenchymal swelling, and interlobular and subcapsular fluid accumulation	AP	[26]

AP: acute pancreatitis, CCK: cholecystokinin, and CP: chronic pancreatitis.

**Table 2:** Phytoceuticals effects in acute pancreatitis (animal studies) and their proposed mechanism of action.

Animals/cell type used	Dose, route of administration, duration of the study	AP model	Findings	Ref.
*Artemisinin (sesquiterpene lactone) found in Artemisia annua, fam. Asteraceae and its derivatives*				

Male Wistar rats	50 mg/kg artemisinin	i.p. 20 *μ*g/kg cerulein, 4 inj. at 1 h interval	↑ apoptosis, caspase-3, ↓ necrosis, amylase, pancreatic edema, inflammatory cell infiltration, NF-*κ*B, MPO, MIP-1*α* protein, IL-1*β* mRNA	[89]

Male SD rats	0.70, 1.75 and 3.50 mg/kg artesunate	r.i.BPD 3.5% sodium taurocholate	↑ survival rate ↓ pancreatic necrosis, hemorrhage, amylase, lipase, pancreatic release of IL-1*β* and IL-6	[90]

*Baicalin (flavone glucuronide) found in Scutellaria spp., fam. Lamiaceae*				

SD rats	5%, i.v.b. 10 mg/100 g f.c.i.v.i. 10 mg/h/100 g	r.i.BPD 3.5% sodium taurocholate	↓ renal pathological changes, mortality, renal Bcl-2 protein, serum NO, plasma endotoxin, serum BUN, IL-6, ET-1, TNF-*α*, PLA2. ↑ renal apoptotic indexes, Bax protein	[91]

Male SD rats	5%, i.v.b. 10 mg/100 g f.c.i.v.i. 10 mg/h/100 g	r.i.BPD 3.5% sodium taurocholate	↓ TNF-*α*, P-selectin, ↑ caspase-3 (↑ apoptosis)	[92]

SD rats	5%, i.v.b. 10 mg/100 g f.c.i.v.i. 10 mg/h/100 g	r.i.BPD 3.5% sodium taurocholate	↓ mortality, endotoxin, TNF-*α* ↑ Bax protein, apoptosis index	[93]

SD rats	5%, i.v.b. 10 mg/100 g f.c.i.v.i. 10 mg/h/100 g	r.i.BPD 3.5% sodium taurocholate	↓ mortality, pathological severity scores, plasma endotoxin, serum PLA2, ET-1, NO, P-selectin	[94]

*Crambene (nitrile) found in brussels sprouts and other plants from fam. Cruciferae*				

Female CD-1 mice	i.v.b. 70 mg/kg	i.p. 50 *μ*g/kg cerulein, 12 inj. at 1 h interval (4, 12, 24, 48 hours after pre-trt.)	↓ amylase, pancreatic edema, necrosis (12 h after pre-trt.) ↑ apoptosis, caspase-3, -8, -9	[51]

Male Swiss mice	i.v.b. 70 mg/kg	i.p. 50 *μ*g/kg cerulein, 3/6/10 inj. at 1 h interval (12 hours after pre-trt.)	↑ apoptosis ↓ MCP-1, IL-1*β*,TNF-*α* ↑ IL-10, TGF-*β*1	[95]

*Curcumin (polyphenol/diarylheptanoid) found in Curcuma longa, fam. Zingiberaceae*				

Albino rats	i.p. 50 mg/kg/d × 6 d (pre-AP induction, post-AP induction or no-AP induction, resp.)	250 mg/100 g L-arginine i.p., twice at an interval of 1 h	↓ amylase, lipase, NAP78, protein carbonyls, TNF-*α*, MPO, pancreatic fgl-2 ↑ caspase-3 (apoptosis)	[96]

Male SD rats	i.p. 100 mg/kg	r.i.BPD 5% sodium taurocholate	↓ ascites, amylase, TNF-*α*, TLR4, NF-*κ*B, IL-10	[97]

WA rats	i.g. 100 mg/kg (curcumin in alcohol) 20 d before AP induction and all through the study	r.i.BPD 3% sodium taurocholate	↓ total histopathologic scores, trypsin activity, NF-*κ*B, AP-1, chemokine, TNF-*α*, IL-6, iNOS, NO ↑ caspase-3 activity	[98]

Kun Ming male mice	i.p. 50 mg/kg/d × 6 d	i.p. 50 *μ*g/kg cerulein	↓ pancreas injury, amylase, ALT, AST, TNF-*α*, NF-*κ*B-p65 ↑ PPAR*γ*	[99]

Male SD rats	i.v. 35 mg/kg/h × 6 h	i.v. cerulein (5 *μ*g/kg/h) × 6 h *and* i.g.ethanol diet × 6 wk → i.v. CCK-8 (3 nmol/kg/h) × 6 h	↓ pancreas injury (histologic changes, amylase, trypsin, neutrophil infiltration) ↓ NF-*κ*B (↓ I*κ*B degradation), AP-1, IL-6, TNF-*α*, chemokine KC, iNOS	[100]

Male SD rats	i.p. 20 mg/kg	2 h ischemia → 6 h reperfusion	↓ amylase, HO^*∙*^, NO, TNF-*α*, WBC count; (lung) ↓ iNOS, TNF-*α*, hyperreactivity	[101]

*Embelin (benzoquinone derivative) found in Ardisia japonica, fam. Primulaceae, Embelia ribes Burm, fam. Primulaceae*				

Male SD rats and male Swiss Webster CD-1 mice	s.c. 20 mg/kg × 5 d	i.p. 50 *μ*g/kg cerulein	↓ pancreatic necrosis and ↑ apoptosis in mouse model, ↑ caspase-9, -3, -8 in mouse model	[7]

*Emodin (anthraquinone) found in Rheum spp., fam. Polygonaceae, Rhamnus spp., fam. Rhamnaceae, Fallopia japonica, fam. Polygonaceae*				

Male SD rats	i.v. 2.5 mg/kg q6 h	r.i.BPD 5% sodium taurocholate	↑ pancreatic blood flow	[102]

Male SD rats	i.v. 2.5 mg/kg q6 h	r.i.BPD 5% sodium taurocholate	↓ TXB2 ↑ 6-keto-PGF1*α*, PGE2 ↓ mortality, pancreatic pathologic scoring	[102]

Male SD rats	i.v. 2.5 mg/kg q6 h	r.i.BPD 5% sodium taurocholate	↓ TXB2 ↑ 6-keto-PGF1*α*, PGE2 ↓ mortality, amylase, lipase, ascites, pancreatic edema, inflammation, necrosis, bleeding, microthrombosis	[103]

Male SD rats	i.v. 2.5 mg/kg	r.i.BPD 5% sodium taurocholate	↓ TNF-*α*, IL-6, edema, extravasation, histological score ↑ claudin-5, occludin	[104]

Male SD rats	i.v. 2.5 mg/kg	r.i.BPD 5% sodium taurocholate	↓ TNF-*α*, IL-6, MPO, pulmonary extravasation & edema; ↑ claudin-4, claudin-5, occludin	[105]

Male SD rats	orally 20/40/80 mg/kg × 28 days	r.i.BPD trinitrobenzene sulfonic acid	↓ TGF-*β*1, pancreatic fibrosis, glandular atrophy, collagen, fibronectin, laminin	[106]

SD rats	i.v. 2.5 mg/kg	r.i.BPD 3.5% sodium taurocholate	↑ intestinal transit ↓ TNF-*α*, IL-1*β*, NF-*κ*B-p65	[107]

SD rats	i.v. 2.5 mg/kg	r.i.BPD 3.5% sodium taurocholate	↓ amylase, histological damage, edema, vacuolization, inflammation, necrosis, NF-*κ*B activation, TNF-*α*, IL-6, IL-1*β*, MDA ↑ SOD	[108]

SD rats	i.v. 2.5 mg/kg	r.i.BPD 1.5% sodium deoxycholate	↑ mCD14 & ICAM-3 expression in pMΦs	[109, 110]

SD rats	i.v. 2.5 mg/kg	r.i.BPD 1.5% sodium ursodeoxycholate	↓ amylase, lung edema, pathological changes, serum TNF-*α*, SAP-induced acute lung injury ↑ AQP1 and AQP5 improved blood gases indexes	[111]

SD rats	i.v. 2.5 mg/kg	r.i.BPD 3% sodium cholate	↓ apoptosis of intestinal mucosa cells, translocation of bacteria and endotoxin ↑ serum leptin, intestinal barrier function	[112]

RPA AR42J cells	10 and 20 *μ*M	cerulein (10^−7^ M) + lipoplysaccharide (10 mg/l)	↓ amylase ↑ apoptotic indices & ↓ necrosis (↓ necrosis/apoptosis ratio); ↓ calcium overload in the cytoplasm ↓ ER stress transducers	[113]

*Emodin (EM) + baicalin (BA)/baicalein (BAe)*				

Male SD rats	i.v. 2.5 mg/kg for EM, 20 mg/kg for BA	r.i.BPD 5% sodium taurocholate	↓ amylase, TNF-*α*, IL-6, TLR4 expression in pancreas and lung, pancreatic and pulmonary damage	[44]

Male SD rats	i.v. 2.5 mg/kg/6 h for EM, 20 mg/kg/6 h for BA	r.i.BPD 5% sodium taurocholate	↓ mortality, ascites, pancreatic pathological scores, amylase, TNF-*α*, IL-6	[114]

*Emodin (EM) + EEN*				

Male Wistar rats	EM (3.0 mg/100 g) by enteral tube q10 h × 6 times	r.i.BPD 5% sodium taurocholate	↓ amylase, ALT, AST, MDA, hepatic & pancreatic MPO, TNF-*α*, AngII, CRP, endotoxin, lactate, mortality, pathological changes, ascites	[115]

*Hesperidin (flavone) found in citrus fruits*				

WA rats	0.3 g/kg s.c.	cerulein 50 *μ*g/kg hourly 7 times	↓ amylase, inflammatory infiltrate, edema, ROS burden, NO	[116]

RPA AR42J cells	53.7 *μ*g/L hesperidin	cerulein	↑ cell viability, apoptosis index; ↓ necrosis, LDH released from the cells, ROS generation	[117]

*Honokiol (lignan) found in Magnolia officinalis (Magnoliaceae)*				

RPA AR42J cells	9.07 *μ*g/L honokiol	cerulein	↑ cell viability, apoptosis index; ↓ necrosis, LDH released from the cells	[118]

*Ligustrazine (tetramethylpyrazine) found in Ligusticum wallichii, fam. Umbeliferae*				

Adult SD rats	i.p. 150 mg/kg/day, 3 days 10 min after the first cerulein injection	i.p. 100 *µ*g/kg/h cerulein, every 1 h, 6 times	↓ amylase, pancreatic MPO, TNF-*α*, IL-1*β*, IL-6, p38, Erk ↑ p53, cleaved caspase-3, apoptosis of acinar cells	[119]

SD rats	NP	r.i.BPD 5% sodium taurocholate.	↑ survival rate, 6-keto-PGF1 alpha ↓ LPO, TXB2	[120]

*Magnolol (lignan) found in Magnolia officinalis (Magnoliaceae)*				

RPA AR42J cells	1.49 *μ*g/L magnolol	cerulein	↑ cell viability, apoptosis index; ↓ necrosis, LDH released from the cells, ROS generation	[117]

BALB/c mice	i.v. magnolol immediately after the AP model was reproduced, then at 9, 12, 24 hours after modelling	i.p. cerulein hourly 7 times	↓ amylase, pancreatic histopathologic score ↑ ratio of myeloid/lymphoid dendritic cells, ratio of IL-10/IFN-*γ*	[121]

*Naringin (flavone) found in citrus fruits and tomatoes*				

RPA AR42J cells	53.7 *μ*g/L naringin	cerulein	↑ cell viability, apoptosis index ↓ necrosis, LDH released from the cells, ROS generation	[117, 118]

*Nordihydroguaiaretic acid (lignan) found in creosote bush, Larrea tridentata (Zygophyllaceae)*				

Swiss albino rats	30 mg/kg/h, orally, thrice at 1 h intervals	cerulein	*in plasma*: ↓ amylase; ↓ IGF-1 *in pancreas*: ↓ TBARS; ↑ SOD; ↑ GSH ↓ edema, damage score ↑ heat shock proteins ↓ MPO, NF-*κ*B, TNF-*α*, phosphorylated p38 ↑ apoptotic cells number modulates the posttranslational modifications of histone H3	[122]

*Resveratrol (trans-3,5,4*′*-trihydroxystilbene) found in grapes, peanuts, soy, giant knotweed (Polygonum cuspidatum) beans*				

SD male rats	30 mg/kg b.m. intraperitoneally	r.i.BPD 4% sodium taurocholate	↓ NF-*κ*B, TNF-*α*, IL-8	[123]

Male Wistar rats	10 mg/kg i.p. 30 min pre-trt.	s.c. 3 × 75 *μ*g/kg CCK-8	↓ amylase, lipase, total pancreatic histological damage, edema, acinar vacuolization ↑ catalase	[124]

Male SD rats	10 mg/kg, injected through penal vein 5 min post-trt.	r.i.BPD 4% sodium taurocholate	↓ severity, NF-*κ*B, iNOS in pMΦs	[125]

Male SD rats	i.v. 20 mg/kg, 5 min post-trt.	r.i.BPD 4% sodium taurocholate	↑ SOD ↓ MDA, serum TNF-*α*, ICAM-1 and VCAM-1 expression in the intestine	[126]

Male SD rats	i.p. 10 mg/kg	r.i.BPD 4% sodium taurocholate	↑ Bcl-2 ↓ Bax, caspases-3 expressions in brain, serum Zonula occludens 1 and Myelin basic protein	[127]

Male SD rats	i.v. 20 mg/kg, 10 min after SAP induction	r.i.BPD 4% sodium taurocholate	↓ amylase, MDA, neutrophil infiltration in pancreas ↑ SOD	[128]

*Dihydroresveratrol (trans-3,5,4*′*-trihydroxystilbene) found in grapes, peanuts, soy, giant knotweed (Polygonum cuspidatum) beans*				

SD rats	p.o. 10, 20, or 50 mg/kg/h	i.p. 50 *μ*g/kg/h cerulein, 6 times and lipopolysaccharide 7.5 mg/kg, 1 time	↓ amylase, lung injury, pulmonary levels of TNF-*α*, IL-1*β*, IL-6, NF-*κ*B	[129]

SD rats	NP	i.p. repetitive administration, cerulein 50 *µ*g/kg/h followed LPS 7.5 mg/kg 1 time	↓ amylase, lung injury, NF-*κ*B, MPO	[130]

*Rhein (anthraquinone) found in Rheum spp. and in Senna spp.*				

RPA AR42J cells	479, 119.8, and 29.9 *μ*g/L rhein	cerulein	↑ apoptotic-to-necrotic cell ratio, p53, cytochrome C, caspase-3, Bax/Bcl-2 ratio (dose dependent)	[53]

male SD rats	10 mg/kg rhein (conjugated with HPDM)	r.i.BPD 3% or 5% sodium taurocholate	↓ amylase, MPO, histological damage in pancreas (inflammatory infiltrate, acinar cell vacuolization & necrosis), ↓ IL-6, TNF-*α* (in serum, pancreas, lung)	[131]

RPA AR42J cells	479 *μ*g/L rhein	cerulein	↑ cell viability, apoptosis index; ↓ necrosis, LDH released from the cells, ROS generation	[117]

*Rutin (rhamnoglucoside of quercetin) found in citrics, grapes, black tea, apple skin peels, amalaki (Emblica officinalis)*				

Swiss mice	p.o. 37.5, 75, or 150 mg/kg, after 24, 36, 48, and 60 h of AP induction	i.p. 8% L-arginine hydrochloride 4 g/kg twice	↓ pain, amylase, lipase, CRP, IL-6, pancreatic MPO, edema index, necrosis, MDA, 3-nitrotyrosine ↑ apoptosis, catalase, SOD	[132]

Male albino Wistar rats	p.o. 100 mg/kg/day from the third week	p.o. ethanol (36% of total calories) 5 weeks, i.p. cerulein 20 *µ*g/kg, thrice weekly, last 3 weeks	↓ amylase, IL-1*β*, IL-18, caspase-1, ASC-NLRP3 ↓ MPO, TBARS, lipid hydroperoxides, oxidative stress index ↑ GPx, SOD, CAT	[133]

Male albino Wistar rats	p.o. 100 mg rutin/kg from 31st day till the experimental period	p.o. EtOH (8–12 g/kg/day) and HFD (22% fat) for 90 days	↑ mRNA expressionof CARD ↓ PYD, caspase-1, and TNF-*α* expressions, serum IL-18 and IL-6	[134]

AngII: angiotensin II, AP: acute pancreatitis, AP-1: activator protein-1, AQP: aquaporin, CARD: caspase activation recruitment domain, d.f.: disease-free, DMSO: dimethyl sulfoxide, EAEEN: emodin-assisted early enteral nutrition, EEN: early enteral nutrition, ER: endoplasmic reticulum, f.c.i.v.i.: followed by continuous i.v. infusion of, fgl2: Fibrinogen-Like Protein 2, HPDM = N,N,N′-trimethyl-N′-(4-hydroxy-3-methylbenzyl)-1,3-propane diamine, gr.: groups, i.g.: intragastric, i.p.: intraperitoneal, i.v.: intravenous, i.v.b.: i.v. bolus, iNOS: inducible NO synthase, LPO: lipid peroxide, mCD14: membrane-bound cluster of differentiation 14 protein, MCP-1: monocyte chemoattractant protein-1, MDA: malondialdehyde, MIP-1*α*: Macrophage inflammatory protein-1*α*, MPO: myeloperoxidase, NAP78: neutrophil-activating peptide 78, NO: nitric oxide, NP: not provided, PDTC: pyrrolidine dithiocarbamate, pMΦs: peritoneal macrophages, PYD: pyrin domain of apoptosis-associated speck-like protein, r.i.BPD:retrograde injection into the bilio-pancreatic duct of, resp.: respectively, RPA: rat pancreatic acinar, s.c.: subcutaneous injection, SAP: severe AP, SD: Sprague-Dawley, SS: Sandostatin, TLR-4: Toll-like receptor 4, trt.: treatment, WA: Wistar-Albino.

**Table 3:** Potential mechanisms of action of phytocompounds in pancreatitis.

*Apoptosis*		

↑ apoptosis	↑ apoptosis (index) of pancreatic acinar cells	Artemisinin [89], baicalin [92, 93], crambene [51, 95], embelin [7], emodin [113], hesperidin [117, 118], ligustrazine [119], magnolol [117], naringin [117, 118], nordihydroguaiaretic acid [122], rhein [53, 117], rutin [132]
	↑ Bax protein	Baicalin [91, 93]
	↑ caspase-3	Artemisinin [89], baicalin [92], crambene [51], curcumin [96, 98], embelin [7], ligustrazine [119], rhein [53]
	Cytochrome C	Rhein [53]
	↑ p53	Ligustrazine [119], rhein [53]
	↓ Bcl-2 protein renal	Baicalin [91]
	↓ p38	Ligustrazine [119]
	↑ Bax protein	Baicalin [91, 93]
	↑ Bax/Bcl-2 ratio	Rhein [53]
	↑ caspase-8, -9	Crambene [51], embelin [7]
	↑ renal apoptotic indexes	Baicalin [91]
	↓ calcium overload in the cytoplasm	Emodin [113]
	↓ ER stress transducers	Emodin [113]
	Modulates the posttranslational modifications of histone H3	Nordihydroguaiaretic acid [122]

↓ apoptosis	↑ Bcl-2	Resveratrol [127]
	↑ mRNA expression of CARD	Rutin [134]
	↓ apoptosis of intestinal mucosa cells	Emodin [112]
	↓ Bax	Resveratrol [127]
	↓ caspase-1	Rutin [133, 134]
	↓ caspase-3, -8, -9, -12	Grape seed proanthocyanidins [214]
	↓ caspases-3 expressions in brain	Resveratrol [127]
	↓ PYD	Rutin [134]

*Inflammation*		

Inflammation markers	↓ CRP	Emodin [115], rutin [132]

Inflammation activators	↓ AP-1	Curcumin [98], curcumin [100]
	↓ ASC-NLRP3	Rutin [133]
	↓ Erk	Ligustrazine [119]
	↓ I*κ*B degradation	Curcumin [100]
	↓ MCP-1	Crambene [95]
	↓ MIP-1*α* protein	Artemisinin [89]
	↓ NAP78	Curcumin [96]
	↓ NF-*κ*B (activation)	Artemisinin [89], curcumin [97–100], dihydroresveratrol [129, 130], emodin [107, 108], grape seed proanthocyanidins [214], nordihydroguaiaretic acid [122], resveratrol [123, 125]
	↓ phosphorylated p38	Nordihydroguaiaretic acid [122]
	↓ PKC*α*	Breviscapine [215]
	↓ TLR4 expression in pancreas	Curcumin [97] emodin + baicalin [44]

Inflammation inhibitors	↑ Nrf2	Grape seed proanthocyanidins [214]
	↑ PPAR*γ*	Curcumin [99]
	↑ IL-10	Crambene [95]
	↑ ratio IL-10/ IFN-*γ* & ratio of myeloid/lymphoid dendritic cells	Magnolol [121]

Promoting phagocytosis of apoptotic neutrophils	↑ ICAM-3 and mCD14 expression in pMΦs	Emodin [109, 110]

Inflammation mediators	↓ chemokine	Curcumin [98, 100]
	↓ cytokines	Rutin [133]
	↓ ET-1	Baicalin [91], baicalin [94]
	↓ ICAM-1 and VCAM-1 expression in the intestine	Resveratrol [126]
	↓ IFN-*γ*	Grape seed proanthocyanidins [214]
	↓ IL-18	Rutin [133, 134]
	↓ IL-1*β*	Artemisinin [89], crambene [95], dihydroresveratrol [129], emodin [107, 108], grape seed proanthocyanidins [214], ligustrazine [119], rutin [133]
	↓ IL-6	Baicalin [91], curcumin [98, 100], dihydroresveratrol [129], emodin [104, 105, 108], emodin + baicalin/baicalein [44, 114], ligustrazine [119], rhein [131], rutin [132, 134]
	↓ IL-8	Resveratrol [123]
	↓ PLA2	Baicalin [91, 94]
	↓ P-selectin	Baicalin [92, 94]
	↓ TNF-*α*	Baicalin [91–93], crambene [95], curcumin [96–101], emodin [104, 105, 107, 108, 111, 115], emodin + baicalin/baicalein [44, 114], grape seed proanthocyanidins [214], ligustrazine [119], nordihydroguaiaretic acid [122], resveratrol [123, 126], rhein [131], rutin [134]
	↓ WBC count	Curcumin [101], rutin [134]

*Nitrosative stress*		

Nitrosative enzymes	↓ iNOS	Curcumin [98, 100]
	↓ iNOS in pMΦs	Resveratrol [125]

Nitrosation products	↓ NO	Baicalin [91, 94], curcumin [98, 101], Hesperidin [116]
	↓ 3-nitrotyrosine	Rutin [132]

*Oxidative stress*		

Oxidative enzymes	↓ MPO	Artemisinin [89], curcumin [96], dihydroresveratrol [130], emodin [105, 115], ligustrazine [119], nordihydroguaiaretic acid [122], rhein [131], rutin [132, 133]
	↓ MPO hepatic	Emodin [115]

Protective antioxidant enyzmes/mechanisms	↑ GPx	Rutin [133]
	↑ catalase	Resveratrol [124], rutin [132, 133]
	↑ HO-1	Grape seed proanthocyanidins [214]
	↑ SOD	Emodin [108], nordihydroguaiaretic acid [122], resveratrol [126, 128], rutin [132, 133]
	↑ GSH	Nordihydroguaiaretic acid [122]

Oxidation products	↓ HO^*∙*^	Curcumin [101]
	↓ lipid hydroperoxides	Rutin [133]
	↓ LPO	ligustrazine [120]
	↓ MDA	Emodin [108, 115], resveratrol [126, 128], rutin [132]
	↓ oxidative stress index	Rutin [133]
	↓ protein carbonyls	Curcumin [96]
	↓ ROS generation	Hesperidin [116, 117], magnolol [117], naringin [117, 118], rhein [117]
	↓ TBARS	Nordihydroguaiaretic acid [122], rutin [133]
	↓ pancreatic fgl-2	Curcumin [96]
	↓ Pancreatic inflammation (pancreatic release of IL-1*β* and IL-6)	Artemisinin [90]

Protection against injury	↑ heat shock proteins	Nordihydroguaiaretic acid [122]

Blood flow	↑ pancreatic blood flow	Emodin [102]
	↑ 6-keto-PGF1*α*	Emodin [102, 103], ligustrazine [120]
	↑ PGE2	Emodin [102, 103]
	↓ AngII	Emodin [115]
	↓ TXB2	Emodin [102], emodin [103], ligustrazine [120]

Pancreatic fibrosis	Pancreatic fibrosis Ł ↑ TGF-*β*1	Crambene [95], emodin [106]
	Pancreatic fibrosis Ł ↓ collagen, fibronectin, laminin, pancreatic fibrosis	Emodin [106]

*Lung*		

Lung inflammatory activators	↓ NF-*κ*B	Dihydroresveratrol [129]
	↓ TLR4 expression	Emodin + baicalin/baicalein [44]
	↓ TNF-*α*	Curcumin [101]
	↓ IL-1*β*, IL-6	Dihydroresveratrol [129]
	↓ pulmonary	Dihydroresveratrol [129]
	↓ TNF-*α* lung	Curcumin [101], dihydroresveratrol [129], rhein [131]

Lung nitrosative stress	↓ iNOS lung	Curcumin [101]

Lung function	↓ lung hyperreactivity	Curcumin [101]
	↑ claudin-4	Emodin [105]
	↑ claudin-5	Emodin [104, 105]
	↑ occludin	Emodin [104, 105]
	↑ AQP1 and AQP5 in lung	Emodin [111]

*Bowel*		

Bowel injury	↓ bacteria and endotoxin translocation	Emodin [112]
	↓ ICAM-1 and VCAM-1 expression in the intestine	Resveratrol [126]

## Abbreviations

Akt: Serine/threonine kinase
ALI: Acute lung injury
AP: Acute pancreatitis
AP-1: Activator protein 1
BIR: Baculovirus Inhibitor of apoptosis protein Repeat
CAM: Complementary and alternative medicines
CRP: C reactive protein
DAMP: The damage-associated molecular pattern
DC: Dendritic cell
DCQD: Chinese herbal formulation Da-Cheng-Qi decoction
EEN: Early enteral nutrition
ER: Endoplasmic reticulum
ET: Endothelin
FasL: Fas ligand (TNF superfamily, member 6)
GSH: Glutathione
GSP: Grape seed proanthocyanidins
GSSE: Grape seeds and skin extract
HO: Hemoxygenase
IAP: Inhibitor of apoptosis proteins
IFN-*γ*: Interferon-*γ*
IL: Interleukine
iNOS: Inducible nitric oxide synthase
LPS: Lipopolysaccharide
MCP-1: Monocyte Chemoattractant Protein 1
MIP-1*α*: Macrophage inflammatory protein-1*α*
MPO: Myeloperoxidase
NDGA: Nordihydroguaiaretic acid
NF-*κ*B: Nuclear factor kappa-light-chain-enhancer of activated B cells
NO: Nitric oxide
NOS: Nitric oxide synthase
PAF: Platelet activating factor
PG: Prostaglandin
PI3K: Phosphatidylinositol 3-kinase
PIDD: p53-induced protein with death domain
PLA2: Phospholipase A2
PLC*β*: Phospholipase C*β*
pMΦs: Peritoneal macrophages
PSC: Pancreatic stellate cells
RAIDD: RIP-associated ICH-1/CED-3 homogenous protein with death domain
RANTES: Regulated on Activation, Normal T Cell Expressed and Secreted
RIPK: Receptor-interacting protein kinase
ROS: Reactive oxidative species
SAP: Severe acute pancreatitis
TGF-*β*: Transforming growth factor-*β*
Th: T helper
TLR4: Toll-like receptor 4
TNF: Tumor necrosis factor
TXA2: Thromboxane A2
TXB2: Thromboxane B2.
